# Non-Destructive Testing for Documenting Properties of Structural Concrete for Reuse in New Buildings: A Review

**DOI:** 10.3390/ma17153814

**Published:** 2024-08-02

**Authors:** Lisbeth M. Ottosen, Wolfgang Kunther, Thomas Ingeman-Nielsen, Serkan Karatosun

**Affiliations:** Department of Environment and Resource Technology, DTU Sustain, Brovej, Building 118, Technical University of Denmark, 2800 Lyngby, Denmark; wolku@dtu.dk (W.K.); thin@dtu.dk (T.I.-N.); serkar@dtu.dk (S.K.)

**Keywords:** circular economy, circular construction, recycling, reinforced concrete

## Abstract

Reuse in new buildings of structural concrete components from demolitions holds the potential for avoiding the use of raw materials to produce new components, including cement for new castings. Reuse rates are high in the circular economy; however, reusing structural components requires documentation of the properties to equate the safety of using reused and new components. Yet, there is no structured or recognized way to perform the documentation. This paper discusses a framework for the documentation requirements for structural concrete, stating the need for documenting the mechanical properties, concrete heterogeneity, and corrosion status of the reinforcement. The possibility is explored for documenting the required properties while the components are in the donor building by use of non-destructive test (NDT) methods. Such use of NDT methods is new. A comprehensive literature survey on the indirect literature, where NDT methods are used to demonstrate similar concrete properties though related to other purposes, is conducted. The overall conclusion is that the use of NDT methods has the potential to document the requested properties before reuse. The next steps towards implementation of NDT for documenting the properties of structural concrete components for reuse involve research in combined NDT methods and the development of AI systems for data interpretation.

## 1. Introduction

**Figure 1:**
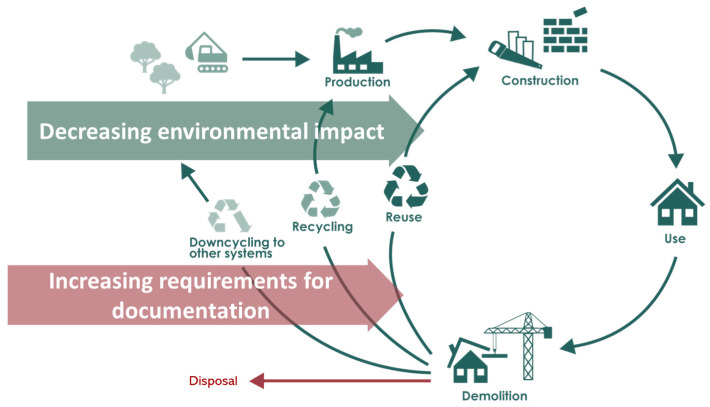
Reuse has potentially less environmental impacts (such as raw material extraction, energy and water use, and CO_2_ emission under production) than recycling/downcycling, but the request for documentation of properties is much higher.

The concrete industry is a major consumer of limited natural resources like water, gravel, sand, and crushed rock. The extraction of natural aggregates, the major constituents in concrete per weight, is often considered an immense natural resource at the global level; however, some regions face a supply constraint due to the overexploitation of natural aggregates in construction [1]. In addition, the raw material consumption to produce cement is equivalent to about 626 kg/per capita, a value higher than the amount of human food consumption [2], and the production of Portland cement comes with about 6% of total anthropogenic greenhouse gases (GHGs) [3]. Thus, concrete production has major environmental impacts, which calls for implementing a circular economy (CE). The transition to CE can involve three strategies: (a) narrowing loops, i.e., using less material input for production to reduce waste output at the end of life; (b) slowing loops, i.e., lengthening the use phase; and (c) closing loops, which can be understood as recycling (recovery or reuse) of the construction materials at end of life [4]. This work focuses on the latter and, more specifically, on the reuse of structural concrete components (beams, columns, load-carrying walls, etc.), i.e., deconstructing the donor building for non-damaged components and reusing them in new buildings, where they are used instead of newly cast components. Such reuse holds the potential for saving more embodied carbon compared with the current recycling of concrete aggregates [5], as illustrated in Figure 1, and can be an important tool in de-escalating resource scarcity. According to the definition of reuse, reusability can be defined as the extent to which the recovered building component in its new life could perform similarly to its earlier life. This paper is focused on the in situ evaluation of the reusability of structural concrete components.

The European Union has set a threshold of a 70% recovery rate for generated waste to more effectively manage construction and demolition waste [6], and better management (compared with the fastest practice where the waste is mixed during the demolition) is required to achieve this threshold. On-site execution and planning of the demolition work highly affect the recovery potential, and the building must be carefully disassembled to increase the recoverability of components and materials. Most of the existing buildings are not designed for such deconstruction. Often, details about the existing buildings are unavailable, and proper guidelines and skills for effective deconstruction do not exist [7]. Based on a fieldwork approach, it was reported [8] that a building element will be recovered for reuse only when the demolition contractor identifies an economic demand for the element, distinguishes appropriate routines to disassemble it, and can control the performance until integration in a new building. Deconstruction can increase the reuse rate; however, there is no available guideline to help practitioners estimate the reuse potential of the building components before deconstruction. Therefore, further research to develop cheap and reliable techniques to investigate the reusability of building components is necessary [7].

The current paper explores whether known NDT techniques can form the base to fulfill the demand for techniques documenting the reusability of concrete components while these components are in the donor building. The total labor costs are considerably higher in selective demolition/deconstruction activities than in conventional demolition, and much more time is needed [9]. To create a business case in CE, it is, therefore, necessary to determine whether the structural components have mechanical properties fulfilling the design criteria for reuse while the elements are still in the donor building. Thus, the NDT methods of interest must be in situ methods, must be fast, and must cover sufficient areas. During inspections before the restoration of houses, minimal disruption is caused during the NDT assessment of the existing structure [10], and a similar inspection could be performed when the donor building is still in use to have sufficient time to plan the selective demolition.

### 1.1. Documentation Is Key to Reusing Structural Components

Today, buildings based on circular principles are niche and they are most often unique demonstration buildings [11,12]. To speed up the transition towards a CE, there is a strong need to bridge the gap from niche to “new normal”. The perception of stakeholders about building component reuse is affected by the potential risks associated with this intervention [7]. A key challenge in choosing the reuse of building components today is the barrier constituted by the lack of standards, certification, and documentation systems for these materials. Such a lack entails a significant documentation burden and economic risk for the building owner when choosing to reuse components [11]. This documentation barrier must be overcome by the development of systems and methods that ensure equality in the choice between reused and new components.

Concerning reuse, each structural component must be considered unique, based on its unique history. Compared with new structural components, the properties of those for reuse may have been reduced during the first use. Documentation may be available for the original design parameters or properties. However, the components for reuse come with a history, and it is still necessary to document the actual properties of the specific structural component at the time of reuse to encompass the possible changes during the first life to ensure that it fulfills the requirements for its new use. Taking core samples from each component is not feasible, neither economically nor technically, because core sampling alone is the same as spot checks and defects might not be identified.

A comprehensive stakeholder analysis of the perception of needs and barriers to construction component reuse [13] highlighted the need for reclaimed components to undergo certain testing to guarantee their quality and safety to reuse them. A procedure should be set out for testing components on their reusability. This test procedure should accommodate the specificity of different components and the condition they are in [13].

### 1.2. Non-Destructive Testing and Reuse

The basic hypothesis motivating this paper is that non-destructive testing (NDT) can form the basis for the documentation of the required properties of structural components of reinforced concrete for reuse. NDT allows one to obtain information on the quality of the material or its durability without damaging the material examined. To our knowledge, such use of NDT methods has not been described previously in the scientific literature. Since there is no directly relevant literature (i.e., the literature on using NDT for the same purpose), this review covers the indirectly relevant literature (i.e., demonstrating that there are other applications of NDT documenting similar concrete properties). The terms for the direct and indirect relevant literature are inspired by [14].

In recent years, NDT has become increasingly essential for reliable and affordable concrete quality control and integrity assessment during the construction of new structures and for the assessment existing structures [15]. Different civil engineering branches already use NDT methods where the material properties are evaluated based on non-contact methods or surface measurements, i.e., no change or damage to the materials inspected. Examples of applications are structural health monitoring (diagnosis and monitoring of structures during their operation) [16], the testing of joints between cast concrete decks [17], and the assessment of fire-exposed concrete [18]. Other disciplines, such as the conservation of historic buildings [19,20] and geotechnical engineering, also use NDT methods [21] to determine internal or subsurface differences in material properties and/or changes over time.

There is a wide spectrum of NDT techniques based on different measurement principles, enabling the production of various sets of information regarding the physical properties of a structure [22]. Compared with coring and destructive tests, NDTs allow the assessment of larger areas/volumes of the structure and give access to information about the spatial variability of material properties of the bulk material and the identification of anomalous areas. Nonetheless, each NDT technique has advantages and disadvantages regarding cost, speed, and accuracy, and the choice of technique should be thoroughly considered concerning the kind of structure to be analyzed and the data to be extracted [23]. In short, non-destructive testing techniques offer interesting methods for the documentation of material properties since they allow relatively rapid mapping of material properties at a moderate cost [24], which is exactly what is needed concerning the documentation of properties before reuse.

### 1.3. Review Aims

This review has four objectives in the process of exploring whether NDT methods originally developed for assessing the integrity and quality of existing concrete structures can form the basis for the documentation of properties of structural concrete components intended for reuse. These objectives are as follows: (1) set overall requirements for the documentation, (2) identify and describe relevant NDT methods, (3) investigate their applicability concerning the requested documentation, and (4) suggest a selection of NDT methods for documenting the essential properties.

## 2. Documentation Requirements

Reusing structural components requires documentation of their properties to fulfill requirements set in the building regulations. Unfortunately, there are currently no standards or guidelines covering the details of the documentation related to the reuse of concrete components.

Before documenting the properties by use of NDT, a visual inspection is advisable to discard the clearly damaged components. Such an initial visual inspection is in line with the common practice of other uses of NDTs in assessments [25]. In Denmark, for example, the environmental screening of buildings is obligatory in connection with reconstruction or demolition to ensure a safe working environment and proper waste handling. If toxic compounds are identified in this process, direct reuse is not a viable option, so the component must be classified as waste and disposed of safely or be decontaminated and used. The visual inspection and the environmental screening should form the basis for the selection of the structural components that are likely to have all properties appropriate for reuse.

The documentation of components for reuse must include mechanical properties and durability status. It is vital to document that the component has no essential degraded or damaged parts from its first use. The documentation must describe both concrete and reinforcement conditions. We suggest that the documentation of reuse potential covers (1) the mechanical properties of the bulk concrete and the dimension and placement of the reinforcement, (2) the presence and location of possible defects/damages in the concrete, and (3) the corrosion status of the reinforcement. In the following Section 2.1, Section 2.2 and Section 2.3, we discuss the requirements within each of these points.

### 2.1. Mechanical Properties

The EN Eurocodes provide design rules for new concrete. Several standards cover the specifications, required physical and mechanical properties, test methods, and classification of newly cast concrete. The test standards are intended for the determination of the material and product properties required for designing buildings and structures. Compressive strength is the key property of concrete. Additional mechanical properties, such as tensile and flexural strength, bond strength, modulus of elasticity, tensile strain capacity, and creep, are necessary to design reinforced concrete components. Quantifying a mechanical property like strength is the highest requirement for assessment (lower ones being ranking or detecting) since values are expected, even with some uncertainties [26].

The documentation of structural components for reuse must include the same properties as requested when using new concrete components. It is also necessary to document reinforcement detailing, i.e., the location and dimensions of the reinforcement, as this is vital for evaluating reuse options. The reinforcement location is important regarding the load-bearing capacity because it determines the level of internal forces and, thus, the resisting bending moment. The concrete cover thickness determines the durability [27] and fire performance [28].

The requirements for the mechanical properties of the components to be reused must be set regarding their use in the new building.

### 2.2. Detect and Locate Heterogeneties

During the first use, parts of the concrete matrix may have been damaged by, e.g., cracking or delamination. In connection with the casting for the first use, heterogeneities such as honeycombing and poor cold joints may have developed for in situ concrete and, to a lower extent, for precast concrete. Such heterogeneities, both from casting and first use, must be identified and evaluated since they strongly influence structural properties and reduce durability, thereby limiting the reuse options. In screening a component for defects and damage, it is sufficient to detect and separate the components or specific areas of the component with heterogeneities from undamaged elements. The screening can also be used to decide where cores are preferably taken in case of doubt about homogeneity.

For new concrete, one or more performance characteristics are typically specified depending on the field of application and the environmental conditions. Conformity control is applied to document that the produced concrete complies with the specified properties. The conformity control is based on a limited number of samples, from which inferences are made for the specimens cast in the same batch [29]. Hence, there is always a risk of making the wrong decision using a structural component with properties that do not fulfill the design requirements [29], even for new components. A conservative approach for conformity control related to components intended for reuse could be based on core sampling. Still, this coring is likely insufficient in numbers (small population) as it is expensive and reduces the value of the component. Additionally, the possible damages are often localized and may not be caught in a random sampling for such conformity control. Therefore, we anticipate the need to develop a new practice and suggest a methodology that involves screening the entire component to identify heterogeneities. The inspection should specify whether the concrete has areas with damage and form a basis for focusing further investigations on these parts if not discharged based on the inspection.

Concrete durability is characterized by its resistance to weathering action, chemical attacks, and other degradation processes [30]. The durability of concrete depends on the resistance it offers to aggression, which can be of physical origin (such as stresses, strains, and temperature) or chemical origin (either from the internal concrete components or from external agents) [30]. When dealing with reuse, assessment of the durability concerning the new use is key. The concrete microstructure plays a fundamental role in durability since the denser the material, the more efficient it is at preventing the transfer of aggressive agents [30]. In aggressive environments, the cover thickness is a crucial design parameter that determines the time until reinforcement corrosion starts. Consequently, any information related to the compactness/porosity of the material microstructure is of interest for diagnostics. The decay of the concrete matrix and reinforcement corrosion is usually triggered by carbonation, chloride ingress, sulfate reactions, alkali–silica reactions, and/or frost. All of these depend on the presence of water [31]. Thus, knowledge of the water content and distribution in the structural component may help evaluate whether the resistance of the component to weathering is decreased by degradation during its first use.

### 2.3. Corrosion Status of the Reinforcement Steel

Tuutti (1982) [32] segments the issue of corrosion into two stages: the initiation stage (penetration of CO_2_ and Cl- through the concrete towards the reinforcement) and the propagation stage (active corrosion). The same two stages can be used in the documentation of structural concrete components for reuse. Reusing as a structural component cannot be recommended if the element exhibits active corrosion. If corrosion is in the initiation stage, the progression of carbonation and chloride ingress must be measured to ensure a sufficient lifetime during the next use concerning the expected exposure conditions. Such progression of harmful substances into the concrete will cause abnormal areas.

### 2.4. Summary of Properties to Be Documented

As discussed in the previous sections, the central reuse properties of structural concrete components are both structural and durability related. This paper suggests that the documentation focuses on three main areas as follows:

(a) Detect and locate heterogeneities.
  - Defects and damages (location and severeness of, e.g., cracks);
  - Moisture status.
(b) Quantify structural properties of the components not failing (a).
  - Compressive strength;
  - Reinforcement detailing.
(c) Determine the corrosion status of the reinforcement if doubt.

## 3. Method of the Literature Search

This review was conducted to investigate whether NDT methods theoretically can provide the documentation requested. The methodology adopted to achieve this review objective had the following three steps: (1) Development of a structured framework for conducting a comprehensive literature review to identify all relevant NDT techniques. (2) Use of this framework to gain an understanding of the measurement principles and output data that can be measured with each method. (3) Discussion of different options for combining NDT methods for documentation of the quality of structural concrete components.

### 3.1. Step 1: Identification of Relevant NDT Methods

The relevant NDT methods were identified through screening. The literature search was finalized on 1 January 2024, using DTU FindIt (https://findit.dtu.dk/), the library database of the Technical University of Denmark, covering approximately 190 million articles from scientific journals and subject databases, 530,000 e-books, and a printed collection of 308,000 books.

A broad search with the search term “non-destructive testing + concrete” resulted in 13,440 hits. Searching for “non-destructive testing + concrete + review”, the hit count was 468 papers. Focusing on journal papers and book chapters in English and published after 1990 reduced the hit count to 407 papers/chapters, which were first screened based on the abstracts. The criteria for the reviews to pass the screening was that they could be related to the use in connection with selective demolition, i.e., they deal with NDT for hardened concrete, use of NDT on existing structures (not limited to lab work), and one-time measurements. Some of the included reviews also contained an experimental section; in these cases, the reviews were only included if the literature review was a substantial part of the paper. With these criteria, 80 reviews passed the screening. These were read in detail and used to identify all relevant NDT methods.

### 3.2. Step 2: Details on the NDT Methods

After identifying the relevant NDT methods in Step 1, the same review papers were used as a setoff for Step 2, describing each method’s measurement principle, measurement process, and output data. Step 2 also included reviewing what the different methods previously were used for related to mechanical properties, concrete heterogeneities, and reinforcement corrosion. In Step 2, additional papers (which were not reviews) were included for the NDT methods to obtain the required information.

### 3.3. Step 3: Combining Methods to Meet the Documentation Requirements

The review papers often suggested combining NDT methods to target a specific property or set of properties. These suggestions were used as a basis for outlining possible combinations of NDT methods to fulfill the requirements for documentation.

## 4. Overview of Identified NDT Methods

The reviews identified during the screening had different scopes. In general, they took offset in either (I) one NDT method (e.g., AC impedance spectroscopy [33]), (II) assessment of heterogeneities (e.g., crack evaluation [34]), or (III) focused on a specific structure (such as a specific bridge [35]).

**Figure 2:**
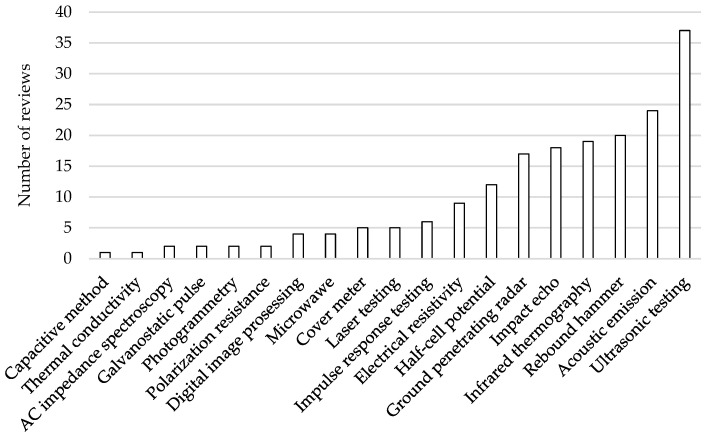
The 19 identified NDT methods from the 80 selected reviews and the number of papers dealing with each method.

Figure 2 shows the 19 NDT methods identified and the number of review papers of the screening which included each of the methods (Step 1). Additional methods were identified, which were not included in this review. These were methods that were based on continuous monitoring. The pull-out test and carbonation test with phenolphthalein were also excluded as not being non-destructive surface methods. Emerging techniques such as sorptivity via surface wettability for estimation of durability [36] were not included if the stage was at laboratory testing.

It is seen from Figure 2 that almost half of the 80 reviews included in the screening included ultrasonic testing. On the other end of the scale, 10 out of the 19 NDT methods were included in 5 or fewer of the 70 reviews. Figure 2 cannot be regarded directly as the scale for, e.g., how often the methods are used in practice or how much research has been conducted on the methods; however, the figure may serve as a rough estimation of these.

## 5. Details in the Different NDT Methods

### 5.1. Basic Principles, Measurement, and Output

To select the appropriate NDT method(s) for a problem and apply it effectively, the operator must (i) have a suitable understanding of the underlying phenomenon, (ii) deploy the testing method(s) correctly, and (iii) apply appropriate and accurate models in the analysis of the collected data to quantify the detected defects or variation of properties [37]. In line with this, the Supplementary Materials Table S1 outlines the basic principles, the measurement procedure, and the expected output for each of the identified methods. The Supplementary Materials Table S1 is an outcome of Step 2 of the review.

The identified NDT methods (Figure 1) can be grouped into seven groups according to the underlying measurement principles as seen in the Supplementary Materials Table S1. The groups are as follows: (I) wave-based techniques (acoustic emission, impact-echo, impulse response method, ultrasonic testing, microwave), (II) electromagnetic wave-based techniques (ground-penetrating radar, covermeter), (III) electrochemical techniques (impedance spectroscopy, electrical resistivity, galvanostatic pulse, half-cell potential, polarization resistance), (IV) thermal methods (infrared thermography, thermal conductivity), (V) camera-based methods (digital imaging, photogrammetry), (VI) laser testing, and (VII) physical methods (rebound hammer).

### 5.2. Field of Use for the Different NDT Methods

The properties to be documented in relation to the reuse of structural concrete components (mechanical properties, heterogeneities, and status of reinforcement) and the identified NDT methods are linked in Table 1. It outlines examples from the literature of the different properties documented or accessed by the 19 NDT methods. International norms and standards, if available, involving the different methods are provided. Table 1 shows the possibilities for use areas identified based on Step 2 of the review. The table can be used as background when narrowing the choice of NDT methods for a specific application or measurement of a specific property.

## 6. Combining Methods to Meet Documentation Requirements

Table 1 shows that there are options to choose NDT methods within all three main areas for the needed documentation. However, it is also seen that there is no single method that can be used in all three areas, so it is necessary to combine methods.

### 6.1. Mechanical Properties

#### 6.1.1. Quantification of Mechanical Properties

Two of the identified NDT methods in Table 1 were reported to be useful in quantifying the concrete’s compressive strength: ultrasonic pulse velocity and rebound hammer. A combination of rebound measurements and ultrasonic pulse velocity measurements are among the most widely used NDT methods regarding concrete strength quantification. The combined use of these two methods is named SonReb [24]. The EN 13791:2019 covers the use of a single relationship between an indirect test method (UPV or rebound hammer) and compressive strength [52]. Measurement on cores is considered to provide the reference strength values, which can be used for comparison or calibration. Some authors state that calibration of SonReb on cores taken from structures is very important, as the use of a direct model without calibration can lead to the wrong evaluation of concrete strength [87]. The NDT methods give a test result from which a strength value can be derived only through a conversion model [88]. The standard EN 13791:2019 provides methods and procedures for the quantification of the compressive strength of concrete in structures and precast components using direct methods (core testing) and indirect methods (NDT–ultra-sonic pulse velocity and rebound hammer). In addition, it establishes the relationship between test results and compressive strength. The standard also includes a guide for assessing the compressive strength class of supplied new structural components in case of doubt if the project owner wishes to validate the specified properties. This guidance could be the starting point for the documentation of the compressive strength related to the reuse of structural components. There may be documentation available on the strength class for the original concrete component; however, whether this documentation is valid for the actual component requires verification. The specific component used for the construction may have had different properties than specified originally, and the compressive strength may have been influenced by degradation during its first lifetime. In other cases, the original strength class of the structural component intended for reuse is unknown. In both cases, the test methodology for documenting the component’s compressive strength can be offset in EN 13791:2019. However, it must be stressed that the standard does not cover the structural components of concrete for reuse, and the degradation issues are not included in the standard; therefore, the methodology described in standard EN 13791:2019 cannot stand alone and must be supplemented by additional documentation.

Many relationships have been proposed to estimate the strength from a couple of (UPV, rebound) value pairs [24]. It appears that no unique relationship exists and that the method must be calibrated for each investigation, as is also the case for the individual methods [30]. When combing the results acquired from at least two NDT methods, a multivariate correlation may be obtained to estimate the compressive strength better. A major advantage of the SonReb method remains its ease of use and low cost [24]. It can be used on any type of structure and concrete, measurements do not require a high level of expertise, and large areas can be investigated relatively quickly [24].

The modulus of elasticity is another design parameter of importance, and also here the ultrasonic pulse velocity can be used as a base and possibly in combination with impact-echo, see Table 1. This option needs to be explored.

The development and validation of a methodology that would, with an acceptable level of confidence, lead to a reliable quantification of strength and elastic modulus remains fundamental to enable the reuse of structural components of concrete. The NDT methods provide encouraging options.

#### 6.1.2. Placement and Dimensions of Reinforcement

The location and dimensions of the rebar must be documented, and the covermeter offers this possibility [70]. Lap splices are unavoidable due to the standardized lengths of commercial rebars. Combined use of a covermeter and ground-penetrating radar can detect a lap splice and estimate the diameter of the two rebars of the lap splice [89]. Thus, available NDT methods can provide detailed knowledge of the placement and cover depth. The next step is to determine whether these offer sufficiently detailed information for documentation related to reuse.

### 6.2. Detect and Locate Heterogeneities

Many types of heterogeneities, such as cracks and delamination, decrease the overall strength and durability of the concrete. EN 13791:2019 covers the quantification of mechanical properties but does not consider the assessment of heterogeneities originating from the first use period. Thus, it is necessary to supplement the quantification with a non-destructive inspection, which involves finding whether the component has defects or abnormalities. Out of the 19 identified NDT methods, 14 can be used to detect and locate different types of heterogeneities in the concrete (see Table 1).

#### 6.2.1. Defects and Damages

When evaluating the potential structural concrete components for possible reuse, it is advantageous to be able to perform a screening when the component is in the donor building aiming at sorting the components into those with heterogeneities and those without. With the application of a covermeter, ground-penetrating radar, or active thermography, large areas can be investigated quickly, enabling a fast overview of the position of reinforcement, the presence of delamination and voids, and the presence and distribution of increased moisture content [90]. Especially detection of subsurface damage in concrete structures by the use of infrared thermography is becoming popular because it is a fast non-contact testing technique capable of rapidly scanning any surface for damages [79], and there is a trend of applying image processing techniques for boosting the productivity of crack detection in structures [91]. Thus, the ongoing general developments of these methods can support a first screening of the structural components related to reuse.

The screening alone may form the basis for sorting out the components with anomalous areas as non-reusable. However, there may be situations where a closer inspection of selected smaller areas of the component is chosen before the final sorting. For such inspection, the relevant methods are impact-echo, impulse response, ground-penetrating radar, AC impedance spectroscopy, electrical resistivity, thermal conductivity, and rebound hammer (Table 1). Each NDT approach has its own merits, and there is no single technology that is capable of identifying all of the complex deterioration phenomena [37,58]. Many structural problems will be best studied by a particular NDT method, depending upon which physical property of the construction material offers the best scheme of reliable defect detection [37]. Non-destructive evaluation methods can be applied alone to evaluate certain defects or can be combined to cover a wider range of testing capabilities in a complementary manner [37]. The combination of NDT methods is currently considered as one of the more relevant ways to improve the quality of the diagnosis of concrete structures and, e.g., ultrasonic surface waves and resistivity or radar methods are highly complementary for the conjugated evaluation of moisture and porosity [92]. The specific method(s) to apply for a more detailed investigation of anomalous areas may be chosen from knowledge of the possible exposure to deleterious environments in the donor building and the findings from the initial screening.

#### 6.2.2. Moisture and Pore Solution

Since all major durability issues depend to some extent on the presence of water, the water content in the concrete cover may indicate whether there are durability issues. The NDT methods, which can be used to assess possible moisture variations in the concrete are AC impedance, ground-penetrating radar, electrical resistivity, and thermal conductivity (see Table 1. Electrical resistivity is very sensitive to different parameters such as the moisture content and the presence of ions in the pore solution, and the application of this technique could differentiate between the healthy and deteriorated zones [58]. Combining the different methods can give more detailed information. For example, the capacitive method could complement the ground-penetrating radar technique and give information about the water content evolution with depth in the structures (from a few millimeters to several centimeters) [58]. However, currently, there are no proven techniques for characterizing moisture characteristics at the steel–concrete interface, for example, the amount, state (liquid/vapor), and spatial distribution [93], and thus the methods cannot be used to evaluate the moisture status specifically at this interphase critical to the corrosion status and thus the overall durability. However, a high water content in the concrete cover should lead to an investigation if active corrosion occurs (see next paragraph).

### 6.3. Corrosion Status of the Reinforcement

#### 6.3.1. Initiation Stage

Reinforcement corrosion is not an issue in dry and protected concrete. If moist areas are detected during the screening (Section 6.2.2), and if a chloride source is present in the vicinity of the concrete, it is necessary to evaluate whether the concrete cover is critically affected by chloride ingress and, if so, the penetration depth must be determined. There are three methods capable of detecting variation in water and chloride content: the electrical resistivity method, the capacitive method, and ground-penetrating radar. These methods show promise for the in situ monitoring of water content and chloride ingress; however, more research is needed to separate the influence of the two properties/processes on the responses [94,95]. For example, [96] concludes that there are very limited studies that discuss and evaluate the combined effect of two or more parameters on ER measurements. The intrusion of water and chloride contamination of the concrete structure has become an evolving area for the application of the ground-penetrating radar technique [69]. In the near future, lab- and mathematical-based contributions based on the ground-penetrating radar are expected to drastically increase the effectiveness of routine mapping contamination of water and chloride in any concrete structures, although such procedures are still not widely accepted by civil engineers [69]. Because low resistivity is related to rapid chloride penetration and a high corrosion rate, the resistivity of a structure exposed to chloride indicates the risk of early corrosion damage [75]. Currently, resistivity techniques are mainly applied as a supplement to other techniques to detect areas of possible corrosion of steel reinforcement. New applications are developed and used also to map the surface of concrete and to detect areas of resistivity contrasts [58]. Currently, no proven techniques exist for in situ characterization of the moisture content, state and distribution, and electrolyte solution composition at the steel–concrete interphase, though these are considered the main factors influencing corrosion [93]. In summary, many promising NDT methods that provide relevant information about properties relevant to the corrosion initiation stage are available, but guidelines and experiences with intelligent and appropriate combinations of methods and further methodological developments in relation to documentation for reuse and quantifying purposes are needed.

#### 6.3.2. Active Corrosion Stage

Detection of active corrosion may likely mean that the component will be discarded. The electrochemical methods aim to evaluate the corrosion activity directly from the electrochemical conditions of the reinforcement and are by far the most suitable for corrosion monitoring in reinforced concrete structures [71]. The four electrochemical methods are AC impedance, galvanostatic pulse, polarization resistance, and half-cell potential (Table 1). The half-cell potential is described is used for determining the corrosion activity of reinforcement steel [72]. Despite being one of the most commonly used techniques for corrosion monitoring in field applications, the half-cell potential technique can only be used as a qualitative test for locating areas with a high corrosion risk. The polarization resistance remains the most important parameter during the corrosion propagation stage as it provides quantitative information on the corrosion rate [72]. However, more fundamental studies are required to convert the polarization resistance into a corrosion rate [72]. Thus, although there is no implemented all-around method that can document the absence of active corrosion, there is a basis for developing such a method.

Some NDT methods can identify the effects of active corrosion. For example, acoustic emission is effective for monitoring and detecting steel corrosion at an early stage, as the received signals differ in case of corrosion from healthy reinforcement. However, more research is required to fully exploit the potential of the method [71]. Ultrasonic signals and ultrasonic techniques effectively relate to the state of reinforcing bars. They may be used to indicate the presence of damage and the exact location and magnitude of the damage when efficiently combined [60]. Rodrigues [72] suggested a procedure including both electrical and other NDT methods (e.g., capacitive technique and ground-penetrating radar) in maintenance actions, first identifying areas with high corrosion risk, followed by an in-depth investigation of active corrosion occurring at the suspected locations. This may also be applied concerning reuse, but it may also be chosen to discharge components in the first place if there is an increased risk that active corrosion occurs.

### 6.4. Data Interpretation and Artificial Intelligence

The development of AI systems when using non-destructive techniques for inspections and documentation for the reuse potential of components would aid the implementation by improving the data interpretation. In general, NDT assessment remains a field for specialists because of the complexity of measurements and post-processing [7]. The development of efficient AI-enabled systems to substitute the human role in non-destructive testing is an emerging topic of considerable interest [97,98]. Augmented reality (AR) technology, for example, has the potential to revolutionize the visualization of bridge deck conditions by providing a human–computer interface for real-time 3D visualization of GPR results [99].

Much research has been devoted to the development of NDT and data processing for better assessment, and the difficulties in correlating the values measured by NDT methods with relevant mechanical properties are well known [24], e.g., even after more than 65 years of use, it is discussed whether the rebound hammer is effective or not in estimating concrete strength [100]. Related to mechanical properties, a main point is that of calibration, i.e., establishing and using a reliable relationship between values measured by NDT methods and the corresponding descriptive property of the element, e.g., strength [24]. However, each new study invariably leads to the development of a new experimental model, and the quest for a universal correlation factor seems endless (even if some research projects still keep this aim) [101]. The use of artificial intelligence (AI), i.e., machine learning (ML) algorithms, to address this challenge is an active research field [16] and has been for quite some time. For example, as early as 1992, Pratt and Sansalone [102] published a paper on the interpretation of impact-echo signals using AI. One of the common challenges within the application of NDT methods is the automation of the interpretation and analysis of the obtained data. With the generation of massive inspection data, algorithms automating the detection help in cutting down efforts and resources for interpretation [103]. Some techniques, such as the laser scanning technique, for example, generate a large amount of data (big data) that is challenging to store, process, and interpret with traditional data management systems. Recent developments in AI/ML and high-performance computing can potentially address these limitations [16].

AI can also play a significant role in non-destructive inspections, and, e.g., image processing can significantly improve the performance of image classification. Recent advances in the field of computer vision arising from the adoption of deep neural networks have resulted in new perspectives for substituting human roles in laborious data interpretation tasks [104]. However, suitable NDT methods must be selected. The definition of specific indicators and parameters descriptive of the decay of structural components is necessary to assign suitable NDT methods for a specific situation [105]. Training sets must be developed for the different NDT methods to enable the identification of all the relevant defects. An example of developing such a training set based on a wall with cast-in defects can be found in [106], where the aim was to detect defects using IE combined with deep learning networks. Another example is Jang et al. [107], who proposed using deep learning to automate the concrete crack detection process with infrared thermography images, where the developed algorithm was experimentally validated using a lab-scale concrete specimen with cracks of various sizes. Ye and Toyama (2022) [104] developed a video content analysis system that can mimic the ability of the human eye to watch a movie of ultrasonic testing wave motion resulting from a scanning pulsed laser and to understand the way waves propagate and interact with any obstacle (flaw) in their path.

Thus, an important next step is to develop AI systems that would increase the quality of the documentation.

## 7. Discussion

### 7.1. Overall Suggestion for Documentation Procedure

Combining NDT methods is currently considered one of the most appropriate ways to improve the quality of the diagnosis of concrete structures [105]; similarly, the documentation of quality related to reuse must offset the combined methods.

We suggest that the documentation follows these steps to be conducted before the deconstruction.

(1) Discard components that are clearly not suitable for reuse, e.g., visually damaged or containing toxic compounds. It may be beneficial (timewise and economically) to perform this first sorting of the components in connection with the pre-demolition audit. A visual inspection for damage can be performed during the same action and possibly by the same person.
(2) Non-destructive inspection for sorting out components with abnormalities and increased corrosion risk. The choice of non-destructive inspection details will depend on the choice of indicators in combination with prior knowledge of the placement of the component in the building (e.g., outdoor or indoor), building details (e.g., age), and the environmental class the building is placed in (e.g., close to the sea). Ground-penetrating radar and active thermography enable the inspection of large areas relatively quickly and can identify abnormalities in the concrete matrix itself and areas with increased moisture content. Thus, these methods are suggested to be investigated for a screening of the components, passing Step (1). A definition of specific indicators and parameters descriptive of unacceptable damage related to reuse is necessary before bringing the methods into this use. Concerning corrosion risk, ground-penetrating radar can be supplemented with electrical conductivity measurements to identify areas with high corrosion risk. We suggest discarding components with high risk, even without investigating if there is active corrosion. If there is a high risk for corrosion, the service life may be limited even though there is no active corrosion at the time of inspection. It is challenging to evaluate the severity of defects and damages related to reuse. This is an area where more knowledge and development are needed to set criteria and design NDT strategies.
(3) Documentation of mechanical properties. The methods and procedures for the quantification of the compressive strength of concrete in structures using a combination of core testing and ultrasonic pulse velocity and rebound hammer (from EN 13791:2019) can be offset for the documentation of the compressive strength for the components that passed Step (2). The number of cores and where to take them for the calibration of the NDT methods needs to be decided upon (including whether coring is needed for each component or if one component can be representative of a population of similar components) and so do the safety margin on the measurements; however, the basis for the documentation is present. Ultrasonic pulse velocity can also be used for the quantification of other mechanical properties, such as the modulus of elasticity. The placement and thickness of reinforcement can be investigated using a covermeter.

With the progress of science and technology, NDT will be more widely used in, and make greater contributions to, the engineering field [108]. This foreseen progress will support and can include the reuse of structural components of concrete. The developments may ensure that the indicators for abnormal areas during the non-destructive inspection will be more detailed and that they can be identified with increasing certainty. In addition, the use of AI supports certainty in data interpretation. Thus, current developments favor the use of NDT to document the quality of concrete elements for reuse.

### 7.2. NDT and Pre-Demolition Audits

Based on the review conducted, we see clear possibilities for using non-destructive methods for documenting the technical reusability of structural components of reinforced concrete. The ability to document the properties when the components are still in the donor building opens new perspectives.

In the European Waste Directive [6], the main objectives for construction and demolition waste are to ensure that CDW is managed in an environmentally sound way and that reaping the full potential of CDW will contribute to the transition to a circular economy. One of the tools to obtain this is pre-demolition audits (also termed waste audits). Such audits are to be carried out before any renovation or demolition project, for any materials to be re-used or recycled, and for hazardous waste. The European Commission provides guides on how to perform such audits [109]. The waste audit aims to deliver a clear idea of the “to-be-demolished” building infrastructure, including estimates of waste materials that will be set free and recommendations for waste management. It is a first step towards recycling and proper waste management. The auditing process aims to deliver the documents that the owner must attach to a demolition or renovation permit application to open a call for tenders [109].

If NDT methods can be developed to provide sufficient documentation for the properties of the concrete components while still being in the donor building (the building to be demolished), such documentation should preferably be carried out in connection with the pre-demolition audit, so this information could flow to designers and architects who wish to use reused components in new buildings or transformation/renovation projects. Such early knowledge of reusable concrete elements has major benefits. Rose and Stegemann [110] describe that, from the demand perspective, designers require information well in advance of the potential purchase, certainty about the quantity and the quality of the items offered, and a wide choice. At present, items are usually offered at the time that they arise as waste rather than in advance. This precludes the opportunity for them to be incorporated into design development. The situation can be changed by in situ documentation of the properties already in the donor building since the needed information can be obtained well in advance of the demolition.

Selective demolition is more costly than ordinary demolition [9]. Already knowing the demand for reused concrete components and which of the components in the donor building has the required quality before the demolition can be a base for targeting the selective demolition. This means that only the reusable components will be taken down, with the caution necessary for keeping the reuse value.

### 7.3. Perspectives and Future Studies for In Situ Documentation of Reusability

The reuse of building components is not yet a mainstream practice [7]. Küpfer et al. [111] compiled 77 examples of the reuse of concrete components in new structures designed between 1967 and 2022 in Europe and the U.S.A. They reported a large diversity of proven implementation techniques, though the knowledge is fragmented. The examples included, e.g., reuse of load-carrying walls from multi-storage donor buildings to buildings with fewer floors and the use of wall elements in dike construction. There were no examples of load-carrying components where the reuse was designed to use the maximum capacity of the components. The use of NDT to quantify the strength of the concrete components can enable such optimal uses of the components, enabling utilization of the full potential. Whether the in-situ documentation in this case needs to be followed up by a verification after demolition to ensure that the properties have not changed is an important question for future research.

On a project level, the implementation of the reuse strategy globally depends on the perceived risks from clients and design teams. Risk perception modification is a slow and intricate process. The development of technical guidance is a necessary step in this direction. The documentation and analysis of precedents must also be pursued to better inform stakeholders of real risks and benefits and vanish this barrier [111]. For this, the development of guidelines on how to document the properties and reusability of the components can be crucial.

After a possible development of in situ documentation for the reusability of concrete components in a donor building, there are new challenges to overcome before streamlining reuse. For example, there is a need for knowledge and know-how related to connection details, both before the deconstruction and for the next use. However, enabling documentation of reusability is an important step toward scaling the reuse of structural components, and thus procedures based on NDT should be developed. Reuse holds a major possibility for significantly reducing the environmental footprint (e.g., +75% climate change impact and +67% ecological load [112]) compared with conventional alternatives, and thus the environmental gain in reusing structural concrete in a building project can be huge.

## 8. Conclusions

The systematic literature review identified 19 relevant NDT methods for documenting properties of concrete elements intended for reuse. The methods can be distributed into seven groups based on the following measurement principles: sonic and ultrasonic wave-based techniques, electromagnetic wave-based techniques, electrochemical techniques, thermal methods, camera-based methods, laser testing, and physical methods. This paper divides the targets for the NDT measurements into (I) identifying abnormalities in the concrete, (II) the status of reinforcement, and (III) mechanical properties. The overall conclusion is that NDT methods are available, which, combined in the right way, in principle can be used to document the requested properties and status of a concrete component for reuse. Quantitative measures for compressive strength are necessary. The standard EN 13791:2019 gives methods and procedures for estimating the compressive strength of concrete in structures and precast concrete elements by combining core testing (for calibration) with ultra-sonic pulse velocity and rebound hammer. This standard could be the offset for the documentation of the compressive strength related to reuse; however, it must be stressed that the standard does not cover reuse and the possible local reduction caused by changes in the component during the first lifetime. The concrete may have suffered from, e.g., cracking and delamination, and the reinforcement from corrosion. It is advisable to combine different NDT methods to have full documentation that the component does not have abnormal areas. Further research is needed to combine NDT methods and develop AI systems for data interpretation. However, it is encouraging that the basis is there for developing procedures for documenting the reusability of reinforced concrete components.

## Figures and Tables

**Table 1:** Standards and examples of what can be measured with each NDT method in each of the three main areas important for the documentation (In bold are the grouping of the methods in accordance to the underlying measurement principles).

	ASTM and CEN Standards	Mechanical Properties	Heterogeneities	Status of Reinforcement
**Wave-based techniques**				
Acoustic emission	ASTM D-4580-23 (2019) [38] ASTM E2983-14 (2019) [39] ISO 16837 (2019) [40]		Delamination [41], fatigue cracks [42], detect and locate cracks in one measurement [23]	
Impact-echo	ASTM C1383-15 (2023) [43]	Stiffness [44], thickness [20,25], elastic modulus [37,45]	Voids [20,37,41], honeycombs [37,41], delamination [20,37,41], bond integrity [37,41], and cracks [20,37]	
Impulse response	ASTM C1740-16 (2016) [46]		Voiding [47,48], delamination [47,48], debonding [47], honeycombing [47], cracking [47,48], depth of alkali–silica reaction [47]	
Ultrasonic pulse velocity	ASTM C597-22 (2023) [49] ASTM D6760-16 (2016) [50] EN 12504-4 (2004) [51] EN 13791 (2019) [52] ISO 1920-7 (2004) [53]	Compressive strength [15,20,24], rebar embedment depth [41], dynamic modulus of elasticity [54], concrete thickness [55], stress state [56]	Homogeneity [57], cracks [41], [37,48], voids [37,41,57], layer thickness [57], delamination [37,48], honey combing [37], thickness of fire-damaged layer [18], locate zones with concrete expansion [58], fatigue damage due to cyclic loads [59], thermal fatigue [59]	Detecting corrosion damage [59,60], debonding of reinforcement bars [37]
Microwave			Defects, flaws [61], water content [61], inhomogeneity [61], surface conditions [62,63]	
**Electromagnetic techniques**				
Ground-penetrating radar	ASTM D4748-10 (2015) [64] ASTM D6432-19 (2019) [65] ASTM D6087-22 (2015) [66] ASTM D6432-19 (2019) [65]	Concrete thickness [37,41], locating steel reinforcement [37], estimating rebar thickness [67], number, position, and distribution of rebars [68]	Embedded objects [16,58], material interfaces [58], internal defects [58], delamination [41,48], voids [20,48], cracks [48], dielectric permittivity [22], water content [69]	Corrosion products [67]
Covermeter		Location of rebar [25,35,70], size of rebar [70], depth of concrete cover [25,70]		
**Electrochemical techniques**				
AC impedance spectroscopy			Microstructure and interfacial properties [33], differentiate the sound and affected zones [58]	Basis for calculating corrosion rate [33]
Galvanostatic pulse				Reinforcement corrosion rate [71,72]
Half-cell potential	ASTM C876-22b (2022) [73]			Locating areas with high corrosion risk [72], corrosion condition [37,70], corrosion rate [72]
Polarization resistance				Corrosion rate [71,72]
Capacitive method			Saturation degree with depth [58]	
Electrical resistivity	ASTM C1876-24 (2024) [74]		Water content [58,75], moisture content [76], cracking state [58], presence of chemical agents [58]	Risk of corrosion [75], Cl penetration depth [72]
**Thermal methods**				
Thermal conductivity			Moisture content [76,77]	Defects [77]
Infrared thermography	ASTM D4788-03 (2022) [78]		Detect defects at surface and below the surface [79], delamination [37,80,81,82], disintegration [37], cracks [37,48,81], and voids [37,48,81]	
**Camera based techniques**				
Digital imaging			Cracks [34], air pockets, discoloration [83], width of multiple cracks simultaneously [45]	
Photogrammetry			2D and 3D models of buildings [84], crack detection and modeling [14], structural deformation [14], thermal defects [84]	
**Laser technique**				
Laser testing			Occurrence and quantification of surface cracks [16]	
**Physical method**				
Rebound hammer	EN 12504-2 (2021) [85], ASTM C805 (2019) [86], ISO 1920-7 (2004) [53]	Compressive strength [68], surface hardness [20]	Uniformity [70]	

## Supplementary Materials

The following supporting information can be downloaded at: https://www.mdpi.com/article/10.3390/ma17153814/s1, Table S1: Basic principles, the measurement and output from the different NDT methods.
