# Supplementary material for: Non-Destructive Testing for Documenting Properties of Structural Concrete for Reuse in New Buildings: A Review
Source: Materials (Basel). 2024 Aug 2;17(15):3814. doi: 10.3390/ma17153814 (PMC11313633; doi:10.3390/ma17153814)
Supplement: Supplementary file 1 [file materials-17-03814-s001.zip › materials-3063899-supplementary.pdf]

*Supplementary Material*

# Overview of NDT methods. Basic principles, the measurement, and output from the measurements

This supplementary material is linked to the review "Reuse of structural concrete in new buildings. Review of NDT for documentation of properties" by Lisbeth M. Ottosen, Wolfgang Kunther, Thomas Ingeman-Nielsen, and Serkan Karatosun. The supplementary material provides additional information on the identified NDT methods in the form of the principle, measurement, and output.

*Table S1: Basic principles, the measurement, and output from the different NDT methods.*

| | Principle in brief | Measurement | Output |
|---|---|---|---|
| **Wave based techniques** | | | |
| Acoustic emission | The phenomenon of acoustic sound generation in structures under stress is called acoustic emission. It is defined as the transient stress waves generated by the rapid energy from localized sources within a structure [1]. The acoustic emission activity is very low in a stable structure [2]. A material under load emits acoustic waves only after a primary load level is exceeded [3]. Under an applied load, stress affects the material and produces local plastic deformation. Load conditions cause concrete to emit energy in elastic waves due | AE monitoring is a passive monitoring technique [1]. The stress produces an elastic wave that travels outward from the source, moving through the body material until it arrives at sensors attached to the surface of the structure [4]. Due to the specific working principles, only active damage can be detected, meaning that the damage needs to occur when the structure is monitored [5] | Typically, the signals collected by the AC censor are characteristic parameters such as amplitude, duration, etc. There are numerous qualitative as well as quantitative ways to interpret these signal parameters or waveforms [3]. An acoustic emission test covers a large area with one test, and it can also be used for continuous monitoring [4]. A computer-based procedure including AE source location, AE event counting from censors, and statistical analysis applied to the AE time series has been developed [6] |

| | | | |
|---|---|---|---|
| | to various material-relevant damage mechanisms [3]. | | |
| Impact-echo | The method is based on the propagation of stress waves in a plate structure. An impact is applied to the surface of test object. The resulting stress wave propagates into the medium and is reflected from the opposite surface (or from a defect in the object). Multiple reflections between the surfaces result in a thickness resonance with a frequency related to the thickness of the object, or the embedment-depth of the defect [7] [8]. | The test involves striking the object with a spherically tipped impactor and measuring the response using a displacement transducer close to the impact location. From the frequency spectrum of the measured displacement time series, the dominant resonance frequency is made and the stress wave velocity, the object thickness is calculated. To obtain the stress-wave velocity, another measurement is performed using two displacement transducers located at a known distance. The velocity is calculated from the difference in the first arrival time in the two measured time series [8]. Measurements are typically collected in a grid across the object of interest. | The directly measured quantities are time series of displacements, from which the stress wave velocity and the dominant resonance frequency may be obtained. Changes in the resonance frequency are indicative of the presence of defects (if object thickness and wave velocity are constant) and may under certain conditions be used to estimate the embedment depth of the defect [9] [10]. The method is used for detailed investigation of the limited part of concrete structure in which internal defects have been already found [11]. |
| Impulse response method | The method is based on the propagation of low-strain, elastic-stress waves [12]. An impact is applied to the test object, and the resulting vibrations measured at a point close to the impact. Changes in the properties of the transfer function, relating the measured response to the applied impact (in frequency domain), is then used | The test involves striking the object with a rubber-tipped, instrumented hammer and measuring the response with a receiver - a direct coupled geophone or an air-coupled microphone. Measurements are typically collected in a grid across the object, with a measurement density reflecting the size and shape of the defect to be investigated [12]. | The method is mainly used for a fast tentative screening of large areas of a structure to determine local areas with possible flaws [11]. The impact force time history is measured at/by the instrumented hammer, and the receiver measures a velocity time series. The velocity frequency response function is calculated from the |

| | | | |
|---|---|---|---|
| | to infer changes in the thickness of or the existence of defects in the test object. | | Fourier transforms of the force and velocity time series. [12] [13]. These properties form the basis of contour plots that are used to delineate anomalous regions [12][13]. |
| Ultrasonic pulse velocity | The ultrasonic pulse velocity (UPV) method uses the ultrasonic sound waves, which are the waves with higher frequency than 20 kHz (35). The working principle of the method is the measurement of the propagation of a pulse of ultrasound waves throughout a distance in concrete. The pulse is transmitted into a concrete element using a transducer in contact with the surface. A gel or grease should be used in the contact area for better coupling between transducer and concrete surface. The propagation velocity of the wave is related to the dynamic modulus of elasticity, Poison's ratio, and density [14] | UPV can be conducted on concrete with three different configurations according to the relative positions of transducers. The most reliable one is placing transducers to opposite sides of concrete, called direct UPV [15]. However, such placement might not always be possible, and the transducers can be placed on adjacent surfaces (semi-direct) or the same surface (in-direct). Steel reinforcement which interferes with UPV measurements [16] and the locations of reinforcements should be determined before UPV measurement to account for this. | The velocity of the ultrasonic pulse can be calculated by the time of the transmission and the distance between transducers. Due to the direct relation, UPV measurement can be used to calculate dynamic modulus of elasticity. Additionally, compressive strength can be estimated using the UPV. Methods like SAFT (Synthetic Aperture Focusing Technique) and tomographic reconstruction can provide high-resolution images of concrete areas, which can be employed for area imaging, duct localization, fault detection, and thickness measurement [45]. |
| Microwave | Microwave NDT relies on the interaction of high-frequency electromagnetic waves with materials, taking advantage of absorption in materials, scattering, attenuation, reflection, transmission, and resonance. These are fundamental effects, and testing attempts to use these in various arrangements for quantitative evaluation of | A unique feature of is that energy is usually coupled into the test medium through air and there is no need for contact. This is done through antennas of various types, open waveguides, and in some cases directly through the fields of transmission lines [17]. Further research is required regarding the application of microwaves in | Can be used as surface, sensor or imaging techniques [19]. It is a new area of research with only a few research articles, which yet have had very limited practical applications [18]. |

| | | | |
|---|---|---|---|
| | properties and conditions in materials [17]. The method uses microwaves and millimeter waves as the source of energy [17]. | terms of radiation safety, the dangers of microwave heating, and other health hazards [18]. | |
| **Electromagnetic techniques** | | | |
| Impulse radar (Ground penetrating radar) | The impulse radar method (often referred to as ground penetrating radar) is a test method based on electromagnetic wave propagation. The propagation speed, attenuation, reflection of wave energy, and changes in polarization of the electromagnetic waves are all controlled by the material properties permittivity, electrical conductivity, and magnetic permeability, as well as changes/contrasts in these properties [20][21]. The method uses short time domain electromagnetic pulses. | A transmitter antenna emits an electromagnetic impulse into the test object. The impulse travels through the object with a velocity depending primarily on the permittivity of the material, and the signal is attenuated due to absorption and geometrical spreading, while part of the energy is reflected at boundaries representing abrupt changes in permittivity (such as cavities, rebars or the opposing face of a slab). A receiver antenna, typically located next to the transmitter antenna, is used to measure the reflected wave train. A series of measurements are obtained on a profile or grid to observe variations in the test object. | The measured signal is a voltage time series representing the amplitude variation of the received wave train as a function of time. Peaks in the observed time series represent reflections from permittivity contrasts encountered. If the permittivity in the medium is known, the timing of the reflection may be used to evaluate the distance to the anomaly. The amplitude of the reflected signal depends on the permittivity contrast(s) encountered, as well as the attenuation and spreading of the signal on its path from transmitter to receiver and may be used to estimate material properties and changes in these. |
| Cover meter | A Cover Meter (or pachometer) is used primarily to measure the presence, size and depth (cover) of ferromagnetic materials (steel, iron) embedded in concrete [4]. It operates by generating an electromagnetic | The instrument consists of two coils on a U-shaped iron cored inductor. An alternating current is applied to one of the cores and generates a primary electromagnetic field [9][22]. The eddy currents induced in the | The measured signal is the voltage induced in the receiver coil and the current applied to the transmitter coil, from which the receiver coil impedance may be calculated. |

| | | | |
|---|---|---|---|
| | field, which induces eddy-currents in the metal structure. These eddy currents generate a secondary electromagnetic field, and a receiver coil measures the combination of the primary and secondary fields. The intensity of the response is a function of the location and size of the embedded metal structure [4][22]. | embedded metal structure produce a secondary magnetic field. The electromotive force induced in the receiver coil is proportional to the field strength of the total field (primary + secondary), and is thus affected by the size, shape and distance to the embedded metal structure [4]. The device is light-weight and portable, and the method is inexpensive and may be used to test large areas quickly [22]. | Most covermeters are calibrated to give directly estimates of the embedment depth and rebar diameter. |
| **Electrochemical techniques** | | | |
| Impedance spectroscopy | Impedance spectroscopy is used to separate conduction processes in inhomogeneous or multiphase materials [23]. It can reveal the microstructure of concrete in high-frequency region, above kilohertz [24]. There are three kinds of paths: (1) continuously connected micro-pores (2), discontinuous pores where the cement paste layers block continuity, and (3), ”insulator" the concrete matirx [24]. The conductivity of the concrete depends on the impedance of all three, which can be incorporated into a circuit model [24]. | An alternating potential is applied between the rebar and a counter electrode, and the resulting current between the rebar and a reference electrode is measured [25]. The resonant frequency of an oscillating circuit between two electrodes are measured [26]. Some simplified physical models have been proposed and the equivalent circuit model can be established based on these physical models. Hereby the electrical properties are used to explain the contribution of solids, liquid and solid-liquid interface to the AC impedance behavior of testing samples [27]. | The impedance spectra are a group of plots representing the measured AC impedance data, among which Nyquist plot is mostly used [27]<br>Some improvements in measurement devices and development of equivalent circuit models for different kinds of binding materials are needed [27]. With some mathematical and physical models, the electrical parameters<br>obtained can be applied to characterize ion migration and steel corrosion [27]. The literature identified in the review all dealt with using the method on the laboratory scale. |

| | | | |
|---|---|---|---|
| Galvanostatic pulse | An anodic current pulse is applied. Upon the interruption of the current pulse, the ohmic drop contribution is immediately lost by the electrode potential and a sudden fall of potential to a value within few microseconds gives the actual polarization of the test specimen [28] | The method is characterized by impressing a small amplitude, short interval anodic current pulse, applied galvanostatically with the help of an external counter electrode over the concrete surface and analyzing the resultant change in potential of the steel reinforcement [28]. | As the passive steel can easily be polarized, non-corroding reinforcement rod shows much higher potential difference than that of a corroding [28]. Thus non-corroded and corroded reinforcement can be distinguished. |
| Half-cell potential method | The half-cell potential (also referred to as corrosion potential) is the open circuit potential of the rebar [25]. The electrical potential difference between the upper steel rebars and a standard portable reference electrode in contact with the concrete surface is measured [29]. The primary purpose is to locate corroding rebars in a concrete structure [30]. | The arrangement of the measurement is a two-electrode configuration. A high impedance voltmeter is required, with the negative terminal connected to the concrete surface and the positive terminal connected to the rebar. A local breakout of the concrete cover is generally required to create a sound contact as the rebar is not readily accessible [31]. | The method does not provide quantitative information on the corrosion rate yet that provides the probability of corrosion. It allows the identification of the main defect points with high corrosion risk [25]. Half-cell potential mapping can help predict the probability of steel corrosion in concrete [32]. The potential of the entire area of inspection is mapped and compared to the relative potential values [30]. |
| Polarization resistance | Polarization resistance is the resistance of the rebar to oxidation during the application of an external potential, i.e., during polarization of the rebar [25]. The linear polarization resistance technique involves applying a small potential sweep on the rebar around its open circuit potential and recording the resulting current. Alternatively, the measurement can be made by applying a current sweep and | Several electrochemical methods have been developed using three- or four-electrode configurations [25]. E.g., in the three-electrode configuration, the electrochemical system consists of a working electrode (the rebar), a reference electrode used for measuring the rebar potential, and a counter electrode that closes the electrical circuit [25]. The concrete resistivity must be determined | Polarization resistance is given by the tangent for zero net current of the potential-current curve obtained by the measurement [25]. The measurement of the polarization resistance only provides an instantaneous estimation of the corrosion rate, which is strongly dependent on the operating conditions [25].<br>The method assumes uniform corrosion, while pitting corrosion is a highly probable |

| | | | |
|---|---|---|---|
| | recording the resulting potential [25]. The polarization (change in potential during reactions) is used to evaluate steel corrosion [33]. | using another technique to compensate for the ohmic drop [25]. | form of steel corrosion in concrete, which might lead to misleading results [33] |
| Capacitive method | The principle of this technique relies on measuring the resonant frequency of an oscillating circuit between two electrodes placed on the concrete surface [26]. | The two metal electrodes form with the surrounding material a dielectric capacitor and the capacity depends on the geometry of the electrodes and on the value of the relative dielectric constant of the material [26].<br>The volume investigated depends on the geometry of the electrodes [34]. | The capacitance value is recovered employing a (high-frequency) resonant circuit supplying alternating voltage. The resonant frequency shift is obtained simply using a frequency analyzer [35]. A calibration allows obtaining the concrete relative permittivity, which is mainly related to the water content and the mixing components [34]. |
| Electrical resistivity | The electrical resistivity is the ability to oppose the passage of an electric current. It is sensitive to a number of parameters related to the nature and condition of the concrete (cement type, aggregates and additions, porosity, water content, salinity of pore solution, and temperature) [26]. Rebar diameter and spacing, concrete cover depth, the direction of the probe, probe spacing and distance from the rebar also affect the measurements [25], which must be taken into account when planning and evaluating the measurements. | The resistivity of the near-surface concrete is measured using electrodes placed on the concrete surface. At least two electrodes are required, but concrete resistivity is generally measured using the Wenner four-probe method [33] [36], which is the application of a DC or AC current between the two outer electrodes and the measurement of the resulting potential difference between the two inner electrodes [25]. The ratio of voltage to current gives resistance. | Contour plots of the response parameter are used to distinguish intact from anomalous regions [12]. Resistivity mapping shows the most permeable spots within a structure, where chloride penetration may be quickest and thus corrosion rates may be highest [37]. Considerable scatter must be expected with coefficients of variation of 20% as considered normal [37]. Electrical resistivity tomography can be performed, enabling determination of the resistivity at different depths reconstructing the spatial distribution [25]. |

| | | | |
|---|---|---|---|
| **Thermal methods** | | | |
| Thermal conductivity | Heat can be transferred by convection, radiation, and conduction. For solids the main type is the conduction. The heat is transferred from the higher temperature object to lower temperature object through the contact surface. As the moisture content of a material increases, thermal conductivity also increases [38]. A concrete element with honeycomb voids or corrosion-related delamination has lower thermal conductivity [39] | A method used to determine thermal conductivity is to supply a known heat input probe in the material and to measure the rise in temperature at a fixed distance from the heat source using thermocouples or thermistors [38]. | Thermal conductivity changes in an element may be an indication of different defects in concrete or different moisture content. Being independent of the salt content of the porous body is one advantage of thermal conductivity measurements. The thermal conductivity depends on the environmental temperature and the density of the material and there is a difficulty in obtaining reproducible calibration curves [40] and subsequently on the data interpretation. |
| Infrared thermography | All objects emit energy in relation to their absolute temperature, which is the method's base [41]. The fundamental concept of the method is that abnormal temperature profiles indicate potential defects [42]. If a concrete surface is viewed with an infrared camera, it will appear quite uniform if the concrete is free of defects [2]. However, cracks or delamination will cause the surface will heat up faster in these areas and hot spots will be observed in the thermal record [2]. | Specialized scanning cameras are used to capture the emitted heat at any temperature and convert the data into thermal images for further analysis [43]. There are two types of infrared thermography;(I) passive thermography where the images are captured under natural ambient conditions providing information based on heat radiation absorbed from the environment, and (II) active thermography where the object is subjected to external heating so that the hidden defects reveal themselves under the thermal non-equilibrium condition [44] | The emitted and reflected infrared radiation from a target surface or object displays an image as a spectrum [45]. Recent advancements have focused on automated data processing and analysis methods and their deployment onto various platforms [43]. The advances in developing high-end IR cameras can potentially enhance the technique's reliability and accuracy [33]. The measurements can be performed employing drones [46]. |

| | | | |
|---|---|---|---|
| **Camera based techniques** | | | |
| Digital imaging | The defects at concrete surfaces are detected<br>and assessed from the analysis of digital images based on their distinct visual characteristics and/or shapes [47]. | The four general steps are: the acquisition of images, image processing, crack detection, and crack analysis (length, area, orientation, etc.) [48]. Genetic algorithms, artificial neural networks, or machine learning algorithms are characterized by very good detection accuracy [49]. Various image processing techniques are available in the literature [50]. | The method can be used for identifying, e.g., cracks and qualitative investigations [49]. The most accurate methods of crack detection on cement composite surfaces are characterized by an accuracy of more than 95% [49]. |
| Photogrammetry | A technique used to create a 3D model (location, size, and depth) through the measurement and analysis of 2D images. The method uses a digital camera to obtain a set of images, from which a 3D model is created of the object under study [45]. Photogrammetry gives reliable geometrical information about physical objects and the environment through processes of recording, measuring and interpreting photographic images and patterns of electromagnetic radiation [51]. | The method involves obtaining multiple images of the object from different positions [52]. Tie-points are identified in multiple images and used in a triangulation procedure to obtain the relative location of the points with respect to the camera positions, based on which a 3D model is created if a physical scale or distance is needed to obtain a correctly scaled model. | The output is typically a 3D model in the form of a point cloud in 3 dimensions, or a meshed surface, representing the object under study. Geometrically rectified images may be draped on the surface of the 3D model, to allow a better representation of visible features on the object. |
| **Laser technique** | | | |
| Laser testing | Laser scanners are mainly used for development of digital models, detailed documentation, and inspection of large | Light pulses are transmitted from a laser source which can be stationary or mobile (drone). Then, the time of those pulses' | Collecting data on different surrounding points can generate a three-dimensional map, also called “point cloud” [54]. |

| | | | |
|---|---|---|---|
| | structure [53]. Principally, a laser source transmits pulses of light and measures the return time of these pulses from the surrounding objects. | return is measured and converted to distance. | Additionally, laser scanning can be used for defects detections and the occurrence of cracks [55]. Using mobile sources, especially with the development of highly advanced drone systems, is progressing. |
| **Physical method** | | | |
| Rebound hammer | Rebound measurement consists of a direct mechanical solicitation of the structure [12]. Since the hardness, in principle, increases when the porosity decreases and that stiffness and hardness are empirically correlated with strength, the rebound is expected to provide a logical means for strength evaluation [12]. | The rebound hammer springs and sliding mechanical mass provide the impact load. The ratio of hammer mass kinetic energies just before and immediately after impact is measured and the relation between the two is the restitution coefficient (Q-value) relies on the loss of energy due to dissipation [50]. The value is correlated with the hardness of the near-surface concrete. | The relationship in the form of equations and graphs between measured values of rebound index and compressive strength of concrete specimens are provided [50]. Due to its simplicity and low cost, the rebound hammer is the most widely used non-destructive test for concrete [12]. However, caution must be taken since, e.g., the rebound number changes with carbonation [56] |

## References supplementary material


1. Meo, M. Acoustic emission sensors for assessing and monitoring civil infrastructures. In Sensor Technologies for Civil Infrastructures Sensing Hardware and Data Collection Methods for Performance Assessment; Woodhead Publishing Series in Electronic and Optical Materials; Woodhead Publishing: Cambridge, UK, 2014; 1, 159–178.
2. Ohtsu, M. Quantitative AE techniques standardized for concrete structures, *Adv. Mater. Res.* 13–14, **2006**, 183–192.
3. Nair, A.; Cai, C.S. Acoustic emission monitoring of bridges : Review and case studies, 32, **2010**, 1704–1714.
4. Lee, S.; Kalos, N.; Shin, D.H. Non-Destructive Testing Methods in the U. S. For Bridge Inspection and Maintenance, *KSCE Journal of Civil Engineering* 18, **2014**, 1322–1331.

5. Verstrynge, E.; Van Steen, C.; Vandecruys, E.; Wevers, M. Steel corrosion damage monitoring in reinforced concrete structures with the acoustic emission technique: A review, *Constr. Build. Mater*. 349, **2022**, 128732.

6. Lacidogna, G.; Niccolini, G.; Accornero, F.; Carpinteri, A., Acoustic emission wireless monitoring of structures, Acoustic Emission and Related Non-Destructive Evaluation Techniques in the Fracture Mechanics of Concrete. Fundamentals and Applications, M. Ohtsu (ed) Woodhead Publishing Series in Civil and Structural Engineering, 2. Edition, **2021**, 15-40

7. Kim, H.; Kim, D.S. Non-destructive testing and evaluation of civil infrastructures using stress wave propagation, *Adv. Nondestruct. Eval*. 270–273, **2004**, 1616–1621.

8. ASTM C1383-15, Standard Test Method for Measuring the P-Wave Speed and the Thickness of Concrete Plates Using the Impact-Echo Method, **2022**.

9. Mccann, D.M., Forde, M.C. Review of NDT methods in the assessment of concrete and masonry structures, *NDT&E Int*. 34, **2001**, 71–84.

10. Sansalone. M., Impact-Echo: The complete story, *Aci Struct. J*. 94, **1997**, 777–786.

11. Andrzej, M.; Martaa, M. Modern NDT systems for structural integrity examination of concrete bridge structures, *Procedia Eng.* 91, 2014, 418–423.

12. Sajid, S.; Chouinard, L. Impulse response test for condition assessment of concrete: A review, *Constr. Build. Mater*. 211, **2019**, 317–328.

13. Sajid, S.; Taras, A.; Chouinard, L. Defect detection in concrete plates with impulse-response test and statistical pattern recognition, *Mech. Syst. Signal Process*. 161, **2021**, 107948.

14. Candelaria, M.D.E.; Kee, S.H.; Yee, J.J.; Lee, J.W. Effects of saturation levels on the ultrasonic pulse velocities and mechanical properties of concrete, *Materials*, 14, **2021**, 1–23.

15. Amini, K.; Jalalpour, M.; Delatte, N. Advancing concrete strength prediction using non-destructive testing: Development and verification of a generalizable model, *Constr. Build. Mater*. 102, **2016**, 762–768.

16. Fodil, N.; Chemrouk, M.; Ammar, A. The influence of steel reinforcement on ultrasonic pulse velocity measurements in concrete of different strength ranges, *IOP Conf. Ser. Mater. Sci. Eng*. 603, **2019**, 022049.
17. Ida, N. Microwave and Millimeter Wave Sensors for Nondestructive Testing and Evaluation, *Lect. Notes Electr. Eng*. 606, **2019**, 143–161.
18. Wahab, A.; Aziz, M.M.A.; Sam, A.R.M.; You, K.Y.; Bhatti, A.Q.; Kassim, K.A. Review on microwave nondestructive testing techniques and its applications in concrete technology, *Constr. Build. Mater*. 209, **2019**, 135–146.
19. Brinker, K.; Dvorsky, M.; Al Qaseer, M.T.; Zoughi, R. Review of advances in microwave and millimetre-wave NDT&E: Principles and applications. *Phil. Trans. R. Soc*. A 378, **2020**, 20190585.
20. Wai-Lok Lai, W.; Dérobert, X.; Annan, P. A review of Ground Penetrating Radar application in civil engineering: A 30-year journey from Locating and Testing to Imaging and Diagnosis, *NDT E Int*. 96, **2018**, 58–78.
21. Omar, T.; Nehdi, M.L. Condition Assessment of Reinforced Concrete Bridges: Current Practice and Research Challenges, *Infrastructures* 3, **2018**, 1–23.
22. Moshtagh, E.; Massumi, A. Seismic assessment of RC buildings by estimation of effective parameters on seismic behaviour using non-destructive tests, *Struct. Des. Tall. Spec. Build*. 20, **2011**, 816–831.
23. Scuderi, C.A.; Mason, T.O.; Jennings, H.M. Impedance spectra of hydrating cement pastes, *J. Mater. Sci*. 26, **1991**, 349–353.
24. Song, G. Equivalent circuit model for AC electrochemical impedance spectroscopy of concrete, *Cem. Concr. Res*. 30, **2000,** 1723–1730.
25. Rodrigues, R.; Gaboreau, S.; Gance, J.; Ignatiadis, I.; Betelu, S. Reinforced concrete structures: A review of corrosion mechanisms and advances in electrical methods for corrosion monitoring, *Constr. Build. Mater*. 269, **2021**, 121240.
26. Metalssi, O.O.; Godart, B.; Toutlemonde, F. Effectiveness of Nondestructive Methods for the Evaluation of Structures Affected by Internal Swelling Reactions : A Review of Electric, Seismic and Acoustic Methods Based on Laboratory and Site Experiences, *Exp. Tech*. 39, **2015**, 65–76.

27. Hu, X.; Shi, C.; Liu, X.; Zhang, J.; De Schutter, G. A review on microstructural characterization of cement-based materials by AC impedance spectroscopy, *Cem. Concr. Compos*. 100, **2019**, 1–14.
28. Sathiyanarayanan, S.; Natarajan, P.; Saravanan, K.; Srinivasan, S.; Venkatachari, G. Corrosion monitoring of steel in concrete by galvanostatic pulse technique, *Cem. Concr. Compos*. 28, **2006**, 630-637.
29. Sadowski, L. Methodology for assessing the probability of corrosion in concrete structures on the basis of half-cell potential and concrete resistivity measurements, *Sci. World J*. 1, **2013**, 714501.
30. Elsener, B.; Andrade, C.; Gulikers, J.; Polder, R.; Raupach, M. Half-cell potential measurements - potential mapping on reinforced concrete structures, *Mater. Struct*. 36, **2003**, 461–471.
31. Verma, S.K.; Bhadauria, S.S.; Akhtar, S. Monitoring corrosion of steel bars in reinforced concrete structures, *Sci. World J*. 1, **2014**, 957904.
32. Marinier, P.; Isgor, O.P. Model-Assisted Non-destructive Monitoring of Reinforcement Corrosion in Concrete Structures. O. Güneş, Y. Akkaya (Eds.), *Nondestruct. Test. Mater. Struct*., RILEM Book, Springer Netherlands, **2012**, 719–724.
33. Zaki, A.; Chai, H.K.; Aggelis, D.G.; Alver, N. Non-Destructive Evaluation for Corrosion Monitoring in Concrete: A Review and Capability of Acoustic Emission Technique, *Sensor*s 15, **2015**, 19069–19101.
34. Balayssac, J.P.; Laurens, S.; Klysz, G.; Lataste, J.F.; Dérobert, X. Non destructive evaluation of concrete contamination by chloride, *Eur. J. Environ. Civ. Eng*. 15, **2010**, 1073–1084.
35. Dérobert, X.; Iaquinta, J.; Klysz, G.; Balayssac, J.P. Use of capacitive and GPR techniques for the non-destructive evaluation of cover concrete, *NDT&E Int*. 41, **2008**, 44–52.
36. Samson, G.; Deby, F.; Garciaz, J.; Perrin, J. A new methodology for concrete resistivity assessment using the instantaneous polarization response of its metal reinforcement framework, *Constr. Build. Mater*. 187, **2018**, 531–544.
37. Polder, R.B. Test methods for on site measurement of resistivity of concrete - a RILEM TC-154 technical recommendation, *Constr. Build. Mater*. 15, **2001**, 125–131.

38. Kot, P.; Muradov, M.; Gkantou, M.; Kamaris, G.S.; Hashim, K.; Yeboah, D. Recent advancements in non-destructive testing techniques for structural health monitoring, *Appl. Sci.* 11, **2021**, 2750.
39. Weil, G.J. Infrared thermographic techniques. V.M. Malhotra, N.J. Carino (Eds.), *Handb. Nondestruct. Test. Concr*., Second edi, CRC Press, **2003**, 15-1-15–14.
40. Kot, A.; Shaw, P.; Riley, M.; Ali, A.S.; Cotgrave, A. The Feasibility of Using Electromagnetic Waves in Determining Membrane Failure Through Concrete, *Int. J. Civ. Eng*., 15, **2017**, 355–362
41. Weil, G. Remote sensing of voids in large concrete structures: runways, taxiways, bridges, and building walls and roofs, *Infrared Technology and Applications XXIV*, 346, **1998**, 305–316.
42. Rehman, S. K. U.; Ibrahim, Z.; Memon, S. A.; Jameel, M. Nondestructive test methods for concrete bridges: A review. *Constr. Build. Mater*. 107, **2016**, 58-86.
43. Kot, P.; Muradov, M.; Gkantou, M.; Kamaris, G.S.; Hashim, K.; Yeboah, D. Recent advancements in non-destructive testing techniques for structural health monitoring. *Appl. Sci.* 11, **2021**, 2750.
44. Naik, M.; Gaonkar, V.; Hegde, G.; Giri, L.I. Detection of Defects in Concrete Structures by Using Infrared Thermography. S.K. Shukla; S. Chandrasekaran; B.B. Das; S. Kolathayar, (eds) Smart Technologies for Sustainable Development. Lecture Notes in Civil Engineering, 78., **2021**, Springer, Singapore.
45. El Masri, Y.; Rakha, T. A scoping review of non-destructive testing (NDT) techniques in building performance diagnostic inspections, *Constr. Build. Mater*. 265, **2020**, 120542.
46. Rakha, T.; Liberty, A.; Gorodetsky, A.; Kakillioglu, B.; Velipasalar, S. Heat Mapping Drones: An Autonomous Computer- Vision-Based Procedure for Building Envelope Inspection Using Unmanned Aerial Systems ( UAS ), *Technology | Architecture + Design*, 2, **2018**, 30-44.
47. Zhu, Z.; Brilakis, I. Machine Vision-Based Concrete Surface Quality Assessment, *J. Constr. Eng. Manag*. 136, **2010**, 210–218.

48. Hoang, N.D. Detection of surface crack in building structures using image processing technique with an improved otsu method for image thresholding, *Adv. Civ. Eng.* 1, **2018**, 3924120.
49. Szelag, M. Evaluation of cracking patterns in cement composites - from basics to advances: A review, *Materials* 13, **2020**, 2490.
50. Zaki, A.; Murdiansyah, L.; Jusman, Y. Cracks Evaluation of Reinforced Concrete Structure: A Review. *J. Phys. Conf. Ser.* 1783, **2021**, 012091.
51. Lorenzo, H.; Pedro, A.; Armesto, J.; Riveiro, B.; Solla, M.; Higinio, G.-J.; Caamaño, C.; Martínez, J.; Álvarez, MM.; Lagüela, S.; Puente, I.; Díaz-Vilariño, L.; Varela, M. Ten years of applying geomatics to construction engineering in Spain: A review. *Dyna* 79, **2012**, 129-146
52. Janowski, A; Nagrodzka-Godycka, K.; Szulwic, J.; Ziółkowski, P. Remote sensing and photogrammetry techniques in diagnostics of concrete structures, *Comput. Concr*. 18, **2016**, 405–420.
53. Lenda, G.; Uznański, A.; Strach, M.; Lewińska, P. Laser Scanning in Engineering Surveying: Methods of Measurement and Modeling of Structures, *Reports Geod. Geoinformatics*. 100, **2016**, 109–130.
54. Xu, Y.; Hwang, S.; Wang, Q.; Kim, D.; Luo, C.; Yang, J.; Sohn, H. Laser active thermography for debonding detection in FRP retrofitted concrete structures, *NDT E Int*. 114, **2020**, 102285.
55. Scalbi, A.; Olmi, R.; Inglese, G. Evaluation of fractures in a concrete slab by means of laser-spot thermography, *Int. J. Heat Mass Transf.* 141, 2019, 282–293.
56. Aydin, F.; Saribiyik, M. Correlation between Schmidt Hammer and destructive compressions testing for concretes in existing buildings, *Sci. Res. Essays*. 5, **2010**, 1644–1648.
